# Occurrence of Acute Aortic Dissection Twice During the Same Pregnancy

**DOI:** 10.21470/1678-9741-2023-0401

**Published:** 2025-02-11

**Authors:** Qiulin Ran, Bin You

**Affiliations:** 1 Beijing An Zhen Hospital, Beijing, People’s Republic of China; 2 The University of Hong Kong-Shenzhen Hospital, Shenzhen, People’s Republic of China

**Keywords:** Thoracic Aortic, Aortic Dissection, Pregnancy, Marfan Syndrome, Stens, Transplants

## Abstract

Pregnancy-related aortic dissection is an uncommon and serious condition since it poses a risk to the lives of both the mother and the fetus. Here, we describe a pregnant woman who suffered from aortic dissection twice during the same pregnancy and whose fetus was safely delivered following aortic root replacement and thoracic aortic stent-graft implantation.

## INTRODUCTION

**Table:** 

Abbreviations, Acronyms & Symbols	
AOD	= Aortic dissection
ARR	= Aortic root replacement
bpm	= Beats per minute
CTA	= Computed tomography angiography
GWs	= Gestational weeks
MFS	= Marfan syndrome

Due to the danger of disease onset and rapid progression, acute type A aortic dissection (AoD) in patients with Marfan syndrome (MFS) during pregnancy should be treated with active surgical treatment. The key factors contributing to the growth of aortic aneurysms and the development of AoD during pregnancy are changes in hormone levels and hemodynamics. When aortic aneurysms or type A AoD do not affect the distal transverse arch of the aorta, blood circulation in the distal aorta does not need to be stopped during surgery, allowing the fetus to survive and the mother to give birth safely. The American Heart Association and the European Society of Cardiology currently recommend that patients with MFS wait until their maximum aortic diameter is 45-50 mm before undergoing aortic surgery^[1,2]^. However, some individuals still suffer from AoD even when their maximum aortic diameter is < 45 mm^[3]^. Due to hormonal and hemodynamic changes in late pregnancy, the occurrence of aneurysmal dilatation of the aortic root increases the likelihood that individuals could experience the recurrence of AoD in unaffected distal aortic segments. Here, we present the case of a pregnant woman with suspected MFS who acquired type A AoD at 21 gestational weeks (GWs). She maintained her pregnancy following aortic root replacement (ARR), but at 33 weeks, she developed acute type B AoD.

## CASE PRESENTATION

A 26-year-old pregnant woman presented to the emergency department of our hospital at 21 GWs with persistent pain in the lower part of the sternum for seven hours. Dexmedetomidine hydrochloride was given for sedation and analgesia, and progesterone (20 mg) was given to suppress uterine contractions. After preliminary examination, the patient's blood pressure was 150/80 mmHg, her pulse was 114 beats per minute (bpm), her respiratory rate was 20 breaths/minute, and her blood oxygen saturation was 100%. Her weight was 82 kg, her height was 181 cm, and her body mass index was 25.03. The patient had a history of chronic gastritis but no previous pregnancy. The patient's mother had unexpectedly died from sudden discomfort in her 40s, but the cause of death was unknown.

Cardiovascular examination revealed a normal heart rhythm, no aberrant heart sounds, and clear breath sounds in both lungs. She exhibited no jugular venous distension and had significant bilateral dorsalis pedis arteries. The condition of the fetus was normal, the fetal heart rate was 155 bpm, and neither uterine contractions nor vaginal hemorrhage occurred. Blood and coagulation tests were performed. Amylase and cardiac enzyme levels were within acceptable limits. A 12-lead electrocardiogram was unremarkable.

**Fig. 1:**
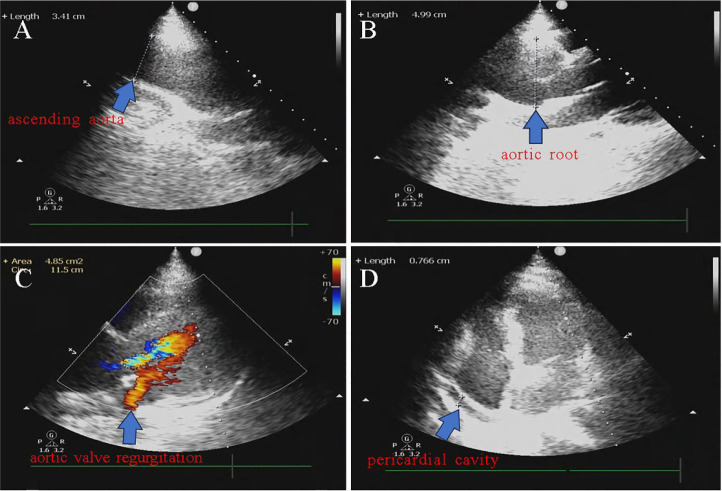
Echocardiography images. A) The inner diameter of the ascending aorta was normal, about 34 mm. B) The maximum inner diameter of the aortic root is approximately 50 mm. C) Moderate regurgitation was displayed in the aortic valve during diastole. D) The pericardial cavity has an 8-mm-deep fluid-filled black region (arrow).

**Fig. 2:**
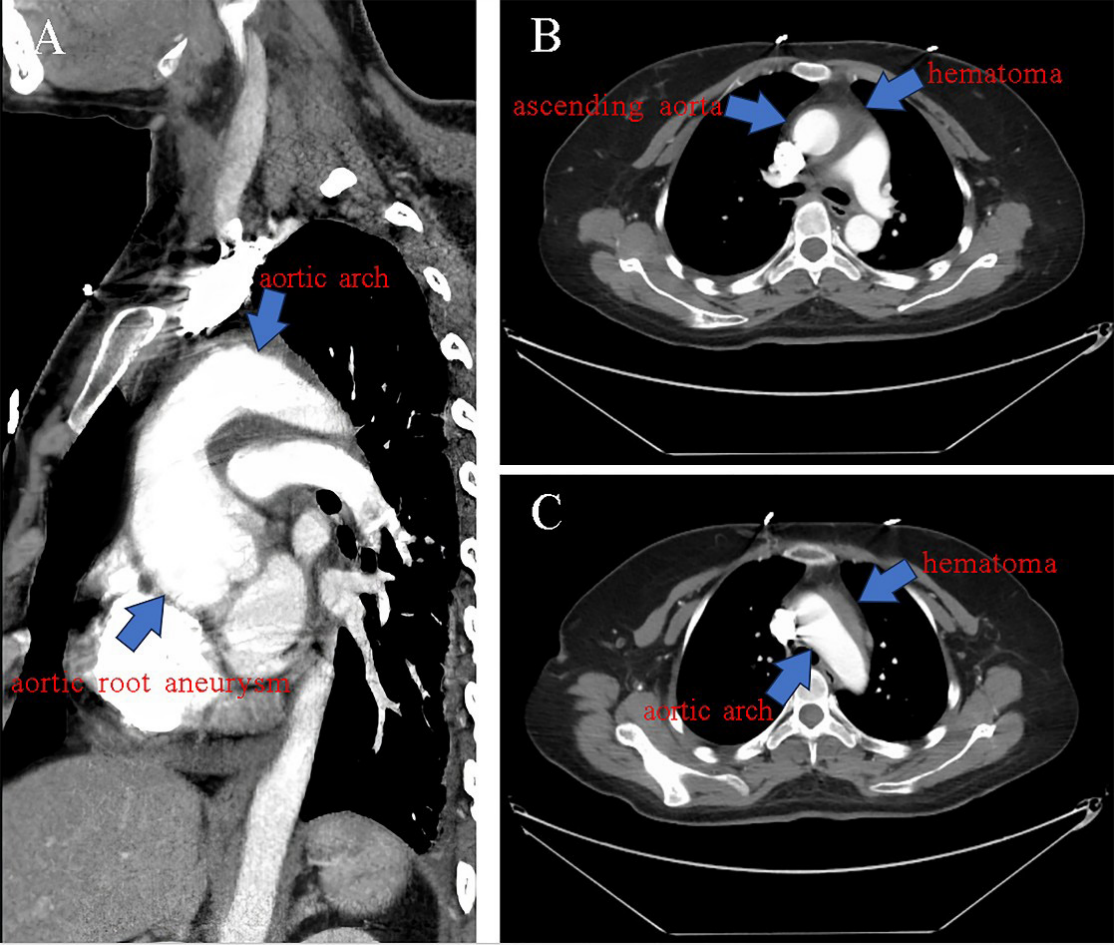
Computed tomography angiography images. A) Aortic root aneurysm (arrow). B, C) Hematoma around the ascending aorta and the aortic arch (arrow).

Transthoracic echocardiography was promptly performed, and the findings revealed that the sinus and root of the aorta were expanded, with a width of 50 mm at the largest point (Figure 1B). The patient had moderate aortic valve regurgitation and mild pericardial effusion (Figure 1C, D). To confirm the diagnosis, the patient's family and herself were advised of the hazards to the fetus and given the option to proceed with computed tomography angiography (CTA). An ascending aortic aneurysm and an intramural hematoma in the ascending and arch portions of the aorta were confirmed (Figure 2A, B, C).

**Fig. 3:**
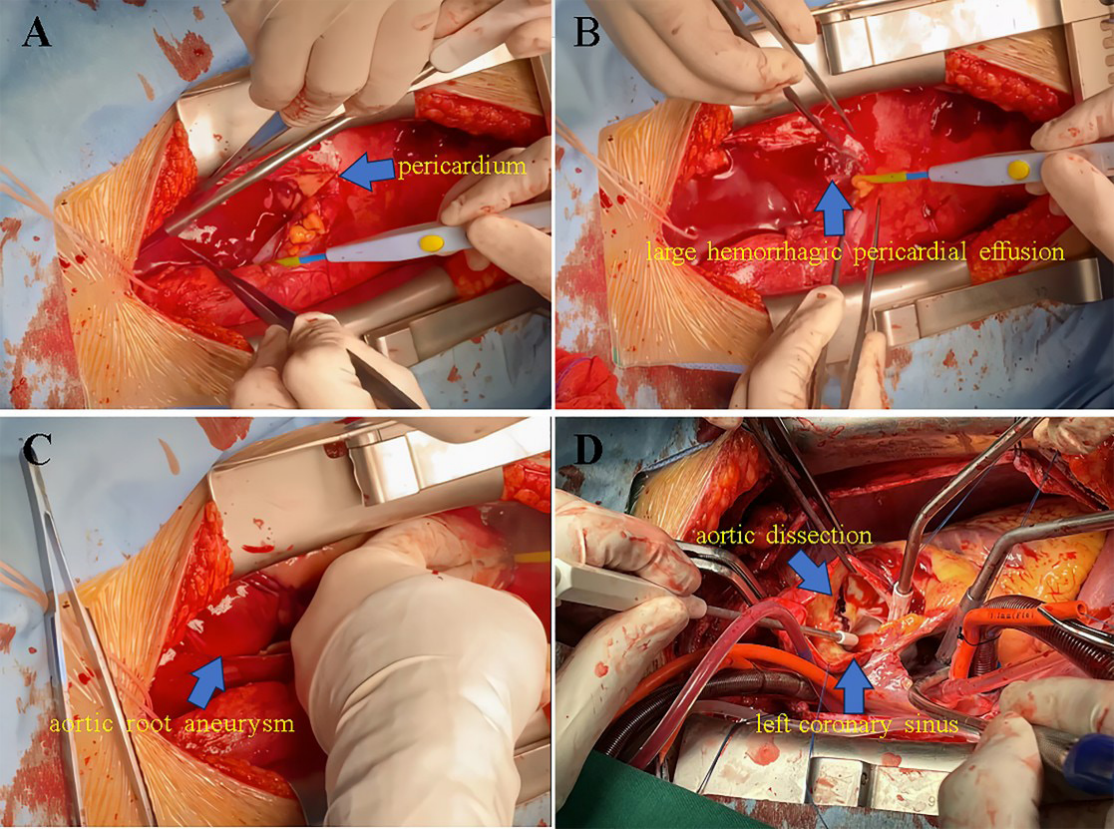
Intraoperative images. A, B) The volume of bloody pericardial effusion was about 300 ml (arrow). C) Aortic root aneurysm (arrow). D) The intimal break of aortic dissection was positioned above the left coronary sinus (arrow).

The patient had thin limbs and severe myopia. She was suspected of having MFS based on the results of the examination and her family history. Hemoglobin levels decreased from 140 g/L to 106 g/L during a routine blood test after the patient was hospitalized in the cardiac intensive care unit, raising concerns about a ruptured aortic aneurysm. Emergency ARR was performed on the patient. Shallow hypothermia was provided via extracorporeal circulation to lessen the effects on the fetus. The volume of bloody pericardial effusion was approximately 300 ml (Figure 3A, B), and the distal ascending aorta was completely blocked. The intimal break was discovered to be positioned above the left coronary sinus after the aorta was incised, and the ascending aorta and aortic arch were involved in AoD (Figure 3D). The valved conduit was sutured with a No. 23 Edwards bioprosthetic valve and a No. 26 Maquet artificial vascular graft, which was used to replace the damaged aortic valve and ascending aorta. The distal end of the artificial vascular graft was anastomosed to the ascending aorta. Finally, the branch of the artificial vascular graft was anastomosed to the brachiocephalic trunk artery. On the first day following surgery, the patient had reduced drainage, and anticoagulants were administered. After recovery, the patient was discharged. β-receptor blockers were used to control blood pressure and heart rate, and the patient's pregnancy continued.

**Fig. 4:**
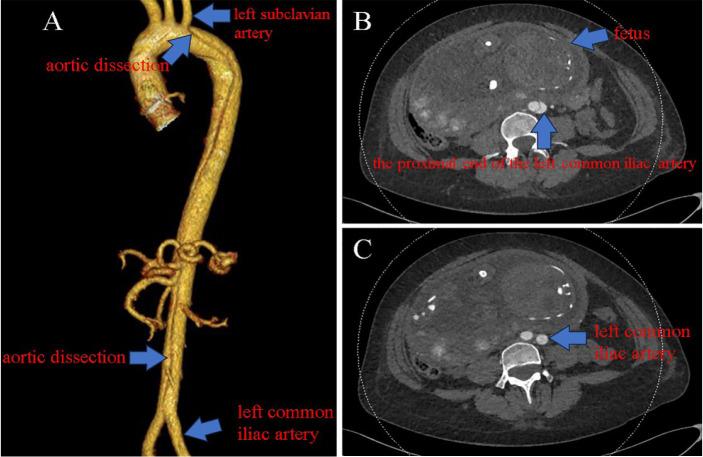
Computed tomography angiography images. A) The aortic dissection starts just proximal to the left subclavian artery and progresses along the descending aorta (arrows). B, C) The aortic dissection only involved the proximal end of the left common iliac artery and did not extend to the distal side (arrows).

The patient returned to our hospital at 33 GWs because of severe back and chest pain. Acute type B AoD was visible on abdominal ultrasonography, and the fetal status was unremarkable. The patient's heart rate was 79 bpm, and her blood pressure was 148/72 mmHg. Before surgery, urapidil was given intravenously to manage her blood pressure, vitamin K1 was given to antagonize warfarin, and tramadol was used for pain relief. CTA of the aorta was performed after alerting the patient and family to the associated dangers. The diameter of the aorta proximal to the left subclavian artery was 26 mm, and the lesion was situated near the start of the descending portion of the aortic arch (Figure 4). After another multidisciplinary discussion before the procedure, we decided to perform thoracic aortic stent-graft implantation, followed by lower uterine segment cesarean section. A live premature baby boy weighing 1800 grams was born after a single-branch aortic stent graft (Castor 282210-2002505) was implanted. His Apgar scores were 1/3, 5/3, and 10/5. Endotracheal intubation and positive pressure ventilation were used to treat the infant in an emergency situation. The infant was then moved to the neonatal critical care unit for further care. Thirteen days following the surgery, the patient was released. Thirty-four days after birth, the baby was successfully released without incident.

## DISCUSSION

Aortic root aneurysm rupture or acute type A AoD are risk factors for MFS patients. The recommended range for the threshold for aortic surgery in MFS patients is currently 45-50 mm^[1,2]^, but some patients still develop AoD even when the maximal aortic diameter is smaller than the recommended range^[3]^. Although most females with MFS who develop AoD have smaller aortic diameters than males, sex differences are not currently considered in the recommendations for elective aortic root surgery^[4]^.

The majority of cases of acute AoD in young women occur during the third trimester of pregnancy and the early postpartum period^[5,6]^. Pregnancy increases blood volume, heart rate, cardiac output, and end-diastolic dimensions. Between 28 and 32 GWs, cardiac output increases by 30%-50%. In addition, the levels of estrogen, progesterone, and relaxin in the body increase, which can inhibit the deposition of collagen and elastic fibers and accelerate the deposition of noncollagen in the artery wall^[7,8]^. Based on congenital anomalies in the medial structure of the aorta in patients with MFS, hemodynamic changes and pathological changes in the aortic wall in pregnant women can lead to an increase in the incidence of AoD^[9]^.

Early diagnosis of MFS and prophylactic ARR can save lives by decreasing the risk of AoD or ruptured aortic root aneurysm^[10]^. There is no need to stop blood circulation in the distal aorta during surgery for aortic aneurysms or type A AoD, which does not involve the distal transverse arch of the aorta, as the pregnancy can be completed, and the fetus can be delivered safely. The ARR in MFS patients does not appear to prevent pregnancy-related type B AoD. Patients with MFS are still at high risk for pregnancy-related type B AoD even after ARR^[11]^. In patients with previous ARR, the distal aorta of the aortic root dilated more progressively. Hemodynamic factors, wall mechanics changes, loss of the Windkessel effect on the descending aorta, or clipping of the aorta during surgery may be related to distal aortic dilatation^[6]^. Therefore, women with MFS who have previously undergone ARR and wish to become pregnant should be informed that they are still at risk of pregnancy-related distal AoD.

In our case, the prepregnancy examination of this patient revealed that the diameter of the aortic root was within the normal range, but the diameter of the ascending aorta had expanded to 51 mm at 21 GWs. Radiation exposure, the extent of vascular lesions, and surgical strategies are directly related to fetal survival. The patient chose to continue her pregnancy after the ARR procedure. At 33 GWs, the patient suffered another acute type B AoD, but the diameter of her aorta at the origin site of the dissection was only 26 mm. Another important consideration in pregnant women with MFS is the absolute diameter of the aortic root and the rate of aortic dilatation^[8]^. In both men and women, the higher the rate of aortic root diameter growth is, the higher the likelihood of acute type A AoD^[4]^. The presence of aneurysmal dilatation of the aortic root indicates that there is still the possibility that the unaffected portion of the distal aorta may redevelop into dissection due to hormonal and hemodynamic changes in the third trimester of pregnancy.

## CONCLUSION

Patients with MFS have an increased risk of acute AoD during pregnancy even after the correction of a type A AoD. The probability of both the mother and child to be saved depends on the gestational age.
